# (−)-Oleocanthal inhibits growth and metastasis by blocking activation of STAT3 in human hepatocellular carcinoma

**DOI:** 10.18632/oncotarget.9782

**Published:** 2016-06-02

**Authors:** Tiemin Pei, Qinghui Meng, Jihua Han, Haobo Sun, Long Li, Ruipeng Song, Boshi Sun, Shangha Pan, Desen Liang, Lianxin Liu

**Affiliations:** ^1^ Key Laboratory of Hepatosplenic Surgery, Ministry of Education, Department of General Surgery, the First Affiliated Hospital of Harbin Medical University, Harbin, China

**Keywords:** hepatocellular carcinoma, (−)-Oleocanthal, tumor growth, tumor metastasis, STAT3

## Abstract

We explored the anti-cancer capacity of (−)-oleocanthal in human hepatocellular carcinoma (HCC). (−)-Oleocanthal inhibited proliferation and cell cycle progression and induced apoptosis in HCC cells *in vitro* and suppressed tumor growth in an orthotopic HCC model. (−)-Oleocanthal also inhibited HCC cell migration and invasion *in vitro* and impeded HCC metastasis in an *in vivo* lung metastasis model. ( )-Oleocanthal acted by inhibiting epithelial-mesenchymal transition (EMT) through downregulation Twist, which is a direct target of STAT3. (−)-Oleocanthal also reduced STAT3 nuclear translocation and DNA binding activity, ultimately downregulating its downstream effectors, including the cell cycle protein Cyclin D1, the anti-apoptotic proteins Bcl-2 and survivin, and the invasion-related protein MMP 2. Overexpression of constitutively active STAT3 partly reversed the anti cancer effects of (−)-oleocanthal, which inhibited STAT3 activation by decreasing the activities of JAK1 and JAK2 and increasing the activity of SHP-1. These data suggest that (−)-oleocanthal may be a promising candidate for HCC treatment.

## INTRODUCTION

Hepatocellular carcinoma (HCC) is fifth most common type of malignant tumor and the third leading cause of cancer-related mortality worldwide [1]. HCC is often diagnosed at an advanced stage when curative therapies are no longer effective. Despite improvements in conventional chemotherapy, the prognosis of HCC remains poor as a result of treatment side effects and drug resistance [2–5]. Therefore, the development of novel effective, non-toxic agents is critical for the prevention and treatment of HCC.

The transcription factor STAT3, a member of STAT family, was initially discovered as an acute-phase response protein related to inflammation [6]. When IL-6 binds to its specific receptor subunit, it can induce dimerization of the gp130 receptor and activation of the gp130-associated Janus kinase (JAK). After that, STAT3 is phosphorylated and undergoes homodimerization, which leads to nuclear translocation, DNA binding, and gene transcription [7, 8]. STAT3 is negatively regulated by many protein tyrosine phosphatases, including members of the SH2-domain-containing tyrosine phosphatase family (SHP-1 and SHP-2) and protein tyrosine phosphatase 1B (PTP-1B) [9–11]. STAT3 is also constitutively active in many kinds of tumors [12, 13], including HCC [14, 15]. This persistent STAT3 activity is thought to contribute to the survival, proliferation, invasion, and angiogenesis of HCC by regulating the expression of target genes [16]. Therefore, STAT3 may be a promising target for HCC therapy [17].

Olive oil is the main source of dietary fat in the Mediterranean diet, consumers of which have a low incidence of chronic inflammatory disease [18]. Higher olive oil intake is also associated with low incidence of various types of cancer, indicating that olive oil may contain potent anti-cancer agents [19, 20]. (−)-Oleocanthal, a phenolic compound in virgin olive oil (VOO), is thought to have anti-oxidative, anti-bacterial, and anti-inflammatory activities [21–25]. It is also effective in treating joint degenerative disease [26, 27]. In addition, recent reports have demonstrated that (−)-oleocanthal has anti-cancer activities in different tumors, including breast carcinoma, prostate carcinoma, and multiple myeloma [28–31]. However, the effects of (−)-oleocanthal on HCC progression are still unclear. The purpose of present study was to examine the effects of (−)-oleocanthal on the growth and metastasis of HCC *in vitro* and *in vivo*.

## RESULTS

### (−)-Oleocanthal inhibits proliferation and cell cycle progression and induces apoptosis in HCC cells *in vitro*

**Figure 1:**
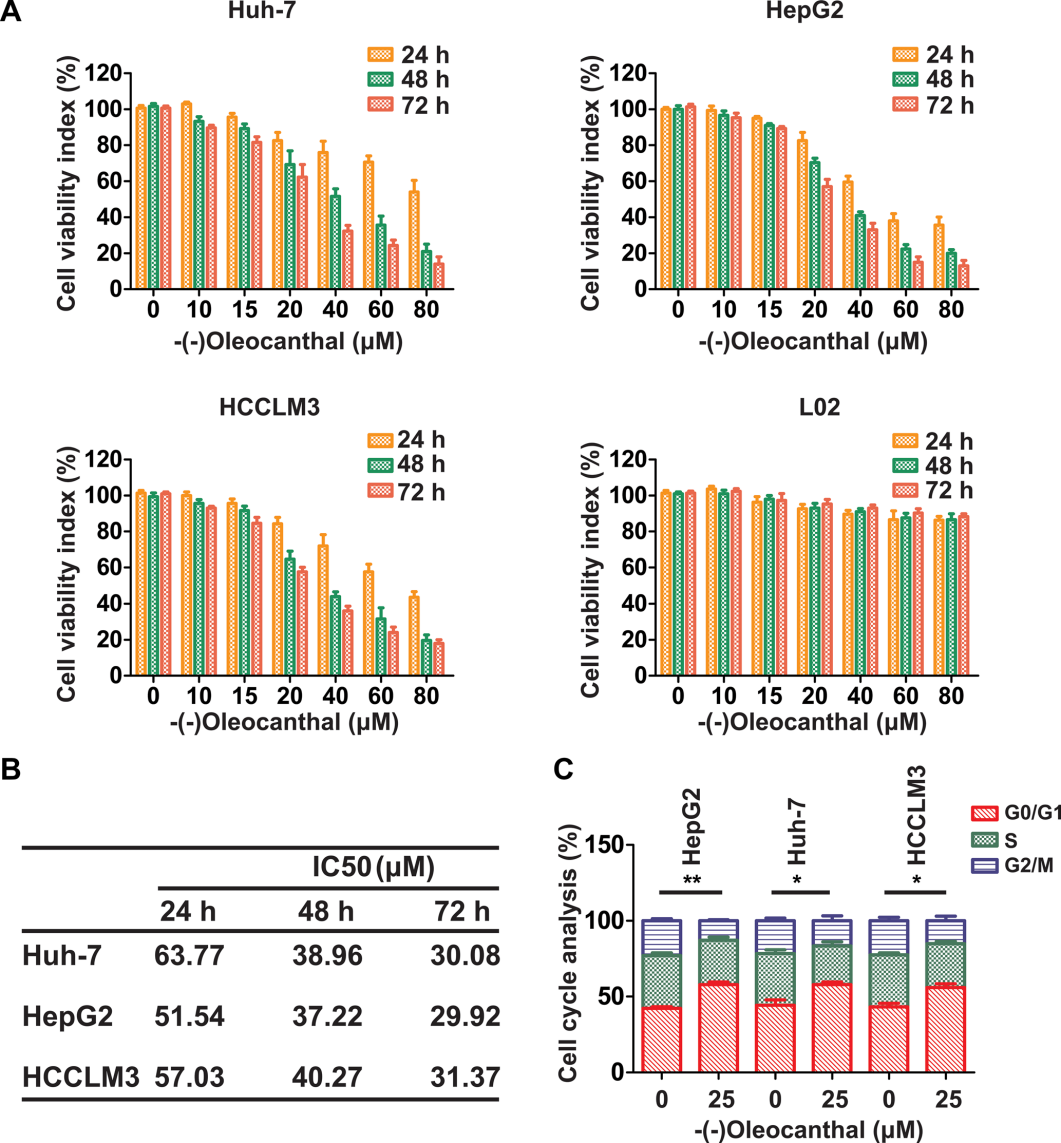
(−)-Oleocanthal inhibits proliferation and induces cell cycle arrest in HCC cells *in vitro* (**A**) HCC cell lines (Huh-7, HepG2 and HCCLM3) and human normal liver cell line (LO2) were incubated with increasing doses of (−)-oleocanthal (0-80 μM) for 24–72 h. Then, CCK-8 assay was performed to investigate the cell viability index. (**B**) The IC50 of (−)-oleocanthal was calculated in the three HCC cell lines. (**C**) Cell cycle analysis in (−)-oleocanthal-treated HCC cells showing arrest in G0/G1 phase. The results represent means ± SD of experiments performed in triplicate. * compared with control, *P* < 0.05. ** compared with control, *P* < 0.01.

**Figure 2:**
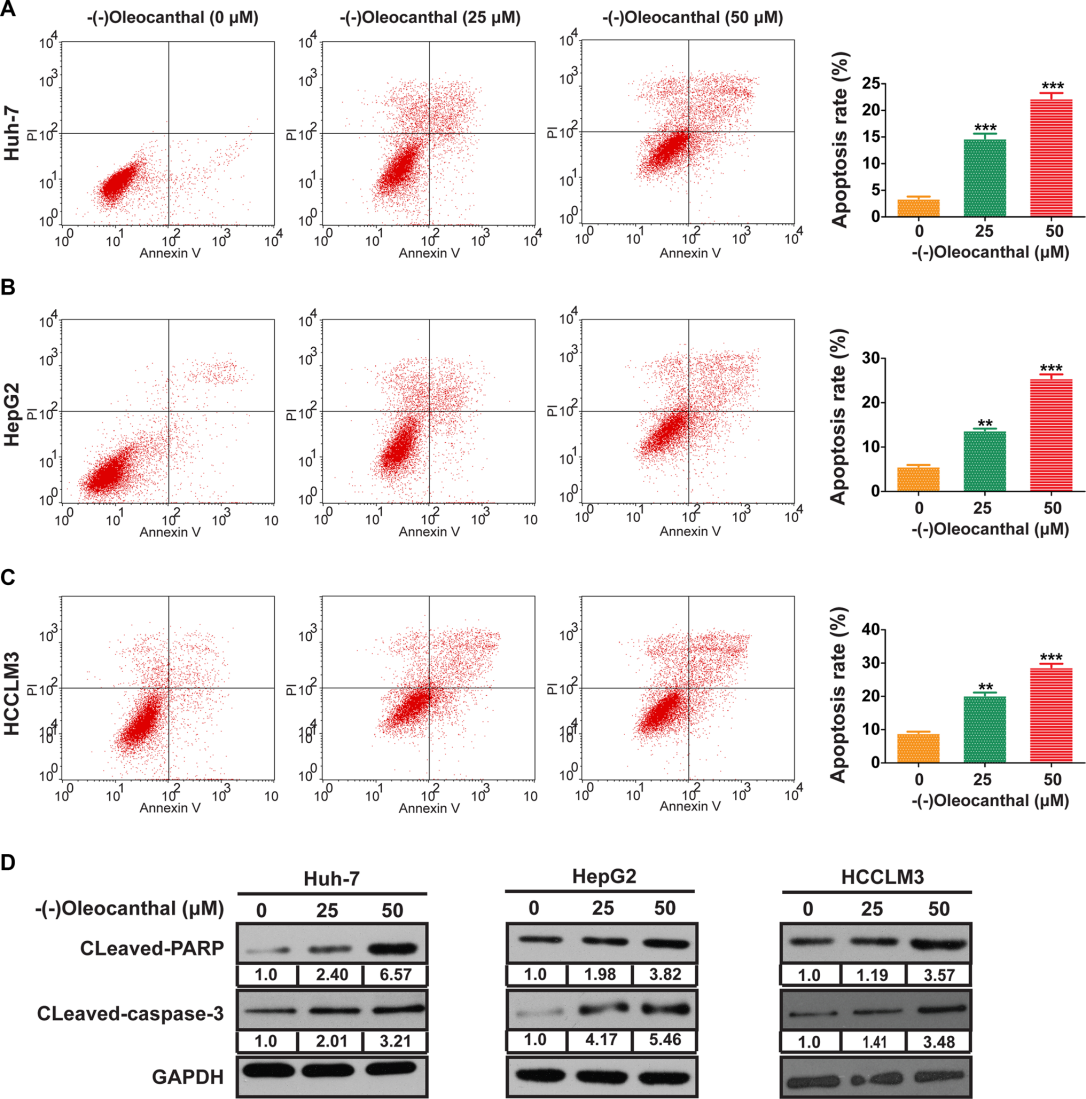
(−)-Oleocanthal induces cell apoptosis in HCC cells *in vitro* (**A–C**) The representative flow cytometry histograms of cell apoptosis for HCC cells treated for 48 h with increased doses of (−)-oleocanthal. (**D**) The expression of cleavages of PARP and caspase-3 was explored by western blotting. GAPDH was used as loading control. Data was presented as the means ± SD of three independent experiments. ** compared with control, *P* < 0.01. *** compared with control, *P* < 0.001.

We first investigated the anti-proliferative activity of (−)-oleocanthal in HCC cell lines (Huh-7, HepG2 and HCCLM3) and a human normal liver cell line (LO2). Cells were incubated with increasing concentrations of (−)-oleocanthal (0–80 μM) for 24–72 h, and the CCK-8 kit assay was then used to evaluate cell viability. (−)-Oleocanthal inhibited HCC cell viability in a dose- and time-dependent manner, but had no effect on LO2 cells (Figure 1A). The IC50 of (−)-oleocanthal is shown in Figure 1B. HepG2 cells were treated with sorafenib as an additional control; the effects of this treatment on cell viability are shown in Supplementary Figure S1. To explore the mechanism by which (−)-oleocanthal suppressed cell viability, flow cytometric analysis was performed to evaluate cell cycle stage and apoptosis in HCC cells after treatment with various concentrations of (−)-oleocanthal. G0/G1 phase arrest increased in HCC cells after (−)-oleocanthal treatment (Figure 1C and Supplementary Figure S2). In addition, (−)-oleocanthal increased the percentage of apoptotic HCC cells in a dose-dependent manner (Figure 2A–2C). (−)-Oleocanthal also increased PARP and caspase-3 cleavage (Figure 2D). These results suggest that (−)-oleocanthal inhibits HCC cell proliferation by inducing cell cycle arrest and apoptosis.

### (−)-Oleocanthal inhibits proliferation and increases apoptosis in an *in vivo* orthotopic HCC tumor model

**Figure 3:**
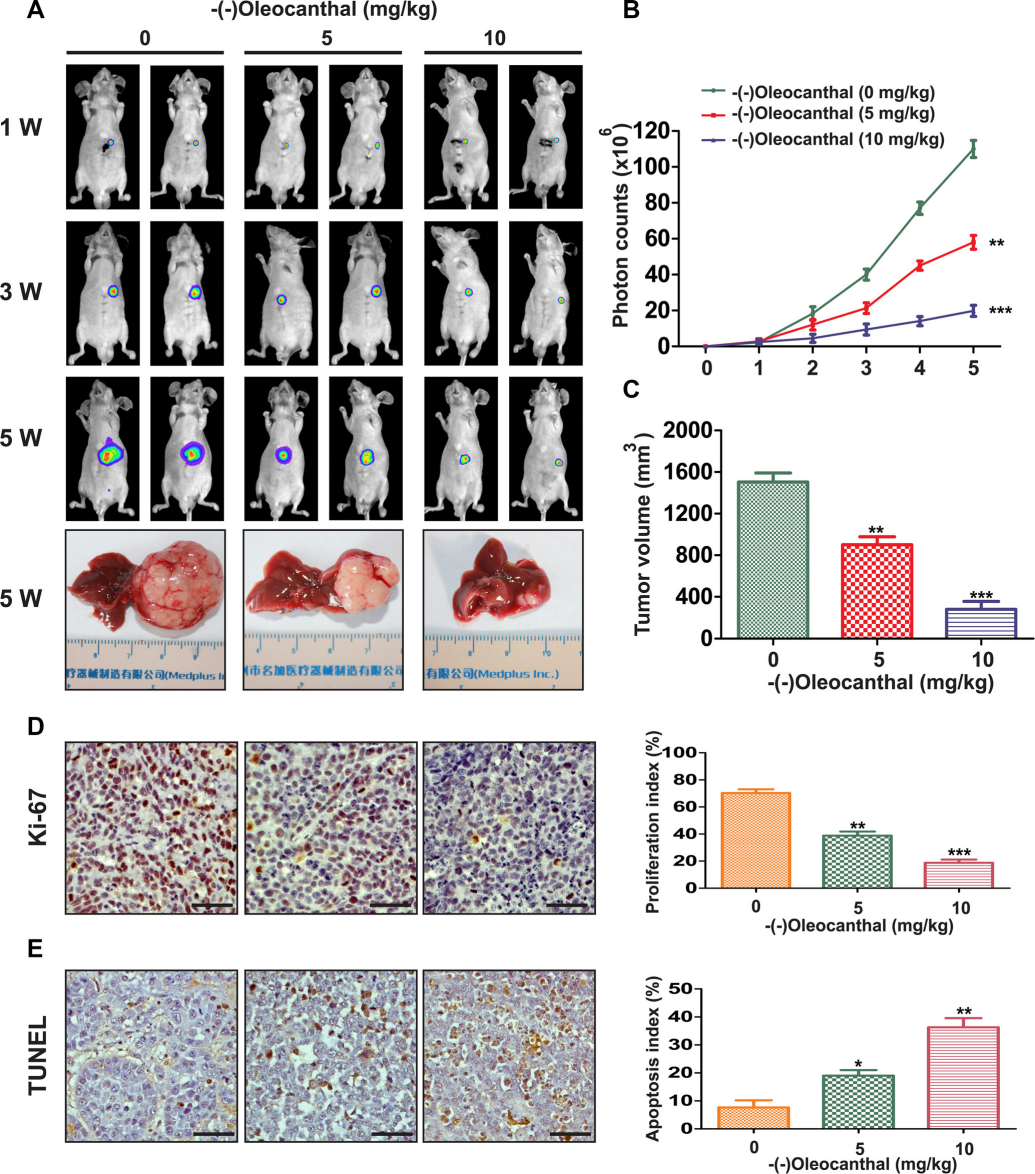
Effects of anti-proliferation and pro-apoptosis by (−)-oleocanthal in an orthotopic tumor model of HCC *in vivo.* (**A**) Representative images of mice from bioluminescent imaging at the first, third and fifth week after (−)-oleocanthal treatment, respectively. The mice were sacrificed at the end of treatment and representative images of gross specimen were shown. (**B**) Quantification of the tumor growth based on the luciferase intensity. (**C**) The tumor volumes were measured with vernier calipers. (**D**) Immunohistochemical analysis of Ki-67 for cell proliferation in tumor tissues. Ki-67-positive cells were counted to evaluate the proliferation index. Scale bars = 200 μm. (**E**) TUNEL analysis was used to detect the apoptosis in tumor tissues. TUNEL-positive cells were counted to calculate the apoptosis index. Scale bars = 200 μm. Data was presented as the means ± SD of three independent experiments. * compared with control, *P* < 0.05. ** compared with control, *P* < 0.01. *** compared with control, *P* < 0.001.

To investigate the anti-tumor effect of (−)-oleocanthal *in vivo*, an orthotopic model was established in mice that were then treated with different concentrations of (−)-oleocanthal for five weeks. Tumor volumes were monitored non-invasively using bioluminescence imaging. Tumor growth was inhibited in the (−)-oleocanthal-treated group compared to the control group (Figure 3A and 3B). At the end of treatment, the mice were sacrificed and tumor volumes were measured using vernier calipers. (−)-Oleocanthal reduced gross tumor specimen sizes (Figure 3A and 3C). To examine the anti-proliferation effects of (−)-oleocanthal *in vivo*, we measured levels of Ki-67, a marker of cell proliferation, in tumor tissues using an immunohistochemistry assay. As shown in Figure 3D, (−)-oleocanthal-treated tumors had fewer Ki-67-positive cells than control group tumors. We then performed a TUNEL assay to determine the effect of (−)-oleocanthal on apoptosis *in vivo*. (−)-Oleocanthal increased the number of apoptotic cells in a dose-dependent manner (Figure 3E). We next investigated the anti-tumor effects of (−)-oleocanthal in orthotopic HCC patient-derived xenografts. High p-STAT3 levels were confirmed in tumors from three HCC patients (HCC-1, HCC-2, and HCC-3) compared to matched non-tumor liver tissues (Supplementary Figure S3A). (−)-Oleocanthal reduced tumor volumes in all three orthotopic HCC xenografts compared to the controls (Supplementary Figure S3B). Characteristics of the three HCC patients are shown in Supplementary Figure S3C.

### (−)-Oleocanthal inhibits HCC migration and invasion *in vitro* and *in vivo*

**Figure 4:**
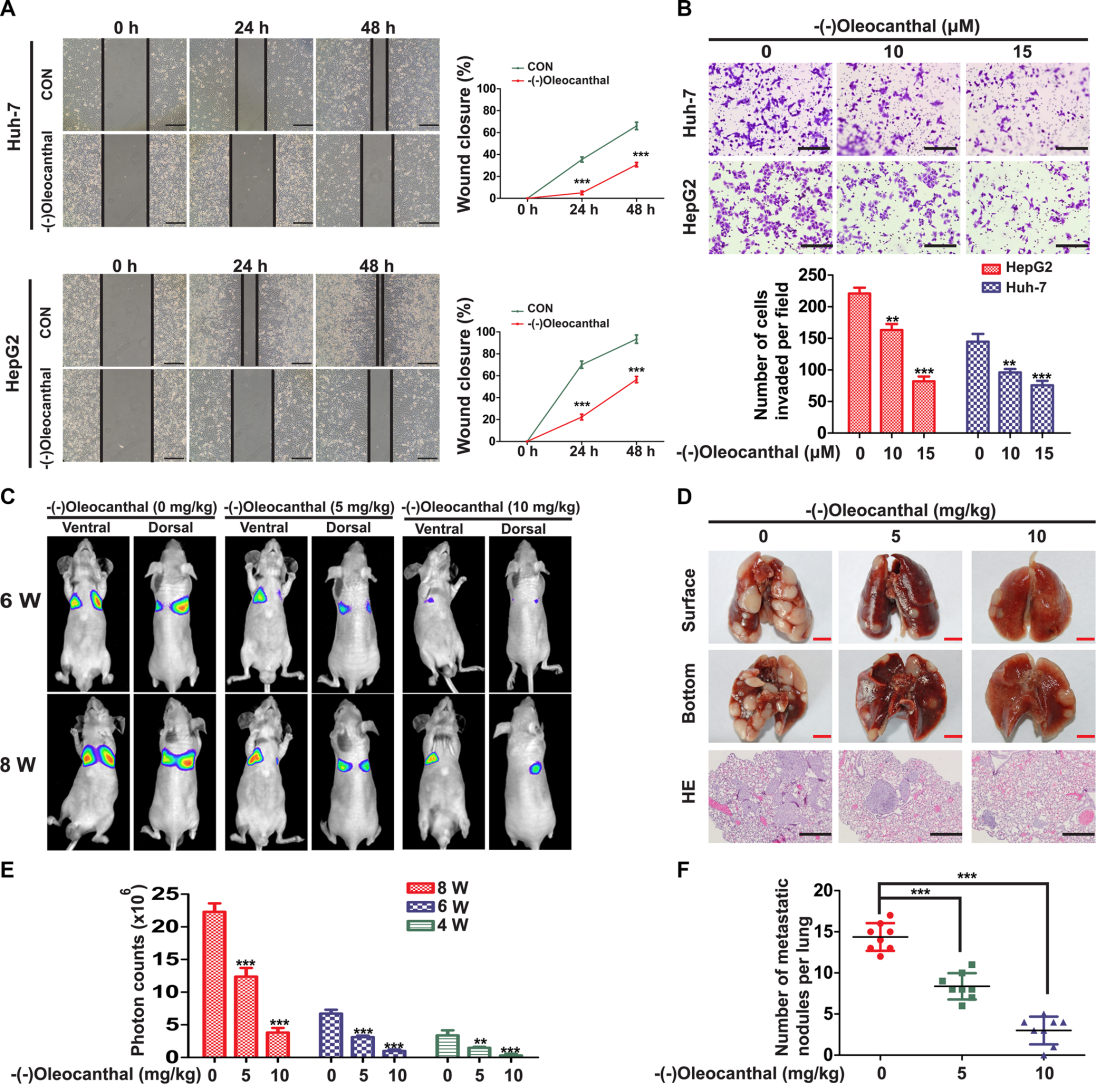
(−)-Oleocanthal inhibits migration and invasion abilities of HCC *in vitro* and *in vivo* (**A**) Representative images of cell migration for Huh-7 and HepG2 cells using wound-healing assay after the treatment with 10 μM of (−)-oleocanthal (left panel). The wound closure was quantified at 24 h and 48 h post-wound by measuring the migrated area (right panel). Scale bar = 50 μm. (**B**) Representative images of invasion assay for Huh-7 and HepG2 cells after the pre-treatment with increasing doses of (−)-oleocanthal for 24 h (top panel). The number of invaded cells was counted (bottom panel). Scale bar = 100 μm. (**C**) Representative images of mice from bioluminescent imaging at the sixth and eighth week, respectively. (**D**) The mice were sacrificed and lungs were excised at the end of treatment. Representative images of gross specimen were shown in the top and middle panel. Hematoxylin and eosin staining of lung tissue samples from the different experimental groups were shown in the bottom panel. Black scale bar = 100 μm. Red scale bar = 0.5 cm. (**E**) Quantification of the tumor growth based on the luciferase intensity. (**F**) Number of metastatic lung foci was detected in each group. Data was presented as the means ± SD of three independent experiments. ** compared with control, *P* < 0.01. *** compared with control, *P* < 0.001.

To explore the effect of (−)-oleocanthal on cell motility, Huh-7 and HepG2 cells were treated with 10 or 15 μM (−)-oleocanthal; these doses did not affect cell viability. (−)-Oleocanthal decreased Huh-7 and HepG2 cell migration ability in a wound-healing assay (Figure 4A). (−)-Oleocanthal also suppressed Huh-7 and HepG2 cell invasion ability in a matrigel-coated transwell assay (Figure 4B). To further investigate the effects of (−)-oleocanthal on HCC *in vivo*, we injected luciferase-expressing HCCLM3 cells into the tail veins of nude mice and monitored tumor metastasis using bioluminescence imaging. Illumination signals were stronger in control group than in the (−)-oleocanthal-treated group (Figure 4C and 4E). At the end of treatment, the mice were sacrificed and lungs were excised to perform hematoxylin and eosin staining. The (−)-oleocanthal-treated group had fewer and smaller lung metastases compared to the control group (Figure 4D and 4F).

### (−)-Oleocanthal suppresses epithelial-mesenchymal transition (EMT) by downregulating Twist expression in HCC

**Figure 5:**
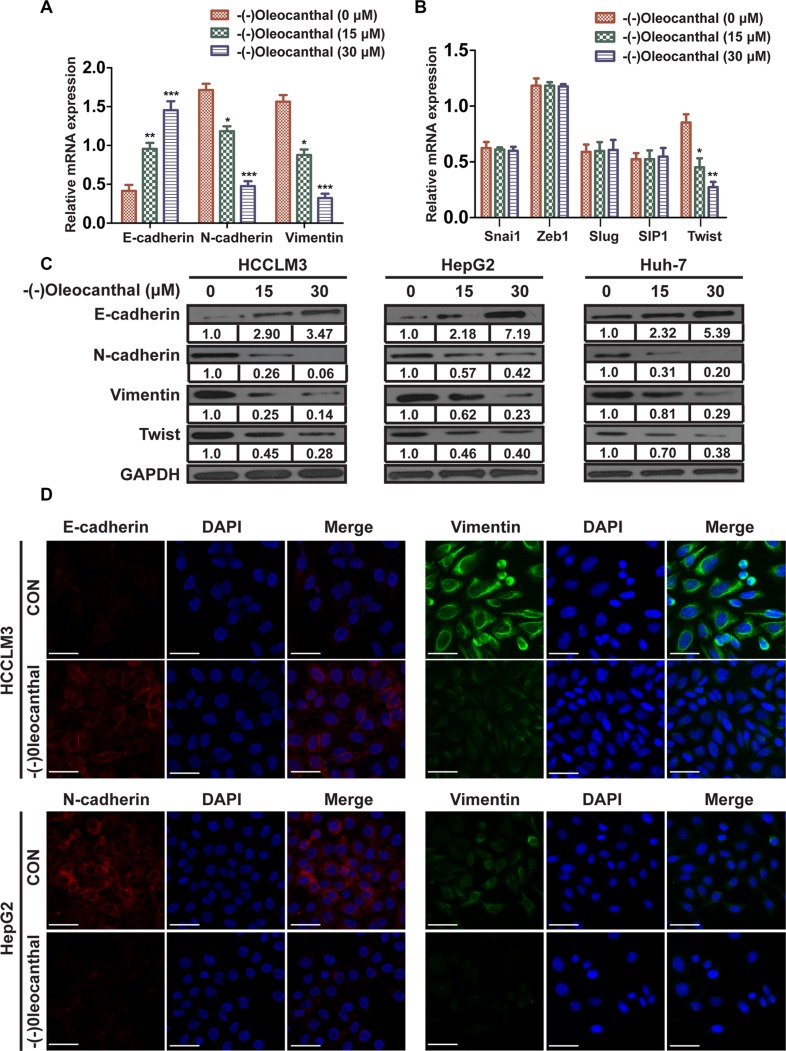
(−)-Oleocanthal suppresses EMT through downregulating the expression of Twist in HCC (**A**) 12 hours after (−)-oleocanthal (15 and 30 μM) treatment, real-time PCR was performed to assess mRNA levels of epithelial marker (E-cadherin) and mesenchymal markers (N-cadherin and vimentin) in HCCLM3 cells. Results were normalized against GAPDH. (**B**) Real-time PCR was performed to assess mRNA levels of several transcription factors including Zeb1, Slug, Snai1, Twist, and SIP1 in HCCLM3 cells. Results were normalized against GAPDH. (**C**) Western blots analysis for the expression of E-cadherin, N-cadherin, vimentin and Twist in HCC cells. GAPDH was used as the internal control. (**D**) Single and merged images were taken to show immunofluorescence staining of E-cadherin (red), N-cadherin (red) and vimentin (green) accompanied by the cell nucleus (blue) stained by DAPI. Scale bar = 50 μm. The results represent means ± SD of experiments performed in triplicate. * compared with control, *P* < 0.05. ** compared with control, *P* < 0.01. *** compared with control, *P* < 0.001.

We next determined the effect of (−)-oleocanthal on EMT. First, real-time PCR was performed to measure EMT marker expression. Expression of the epithelial marker E-cadherin was increased, while expression of the mesenchymal markers N-cadherin and vimentin were decreased, in the (−)-oleocanthal-treated group compared to the control group (Figure 5A). We then measured mRNA levels of the transcription factors Zeb1, Slug, Snai1, Twist, and SIP1, which have been reported to induce EMT by suppressing E-cadherin expression. Real-time PCR analysis showed that (−)-oleocanthal reduced Twist expression, but had no significant effect on mRNA levels of the other transcription factors (Figure 5B). Western blot showed increased E-cadherin and decreased N-cadherin, vimentin, and Twist levels in the (−)-oleocanthal-treated group compared to the control group (Figure 5C). Similar results were observed in the immunofluorescence assay (Figure 5D).

### (−)-Oleocanthal suppresses the transcriptional activity of STAT3 and downregulates the expression of its targets

**Figure 6:**
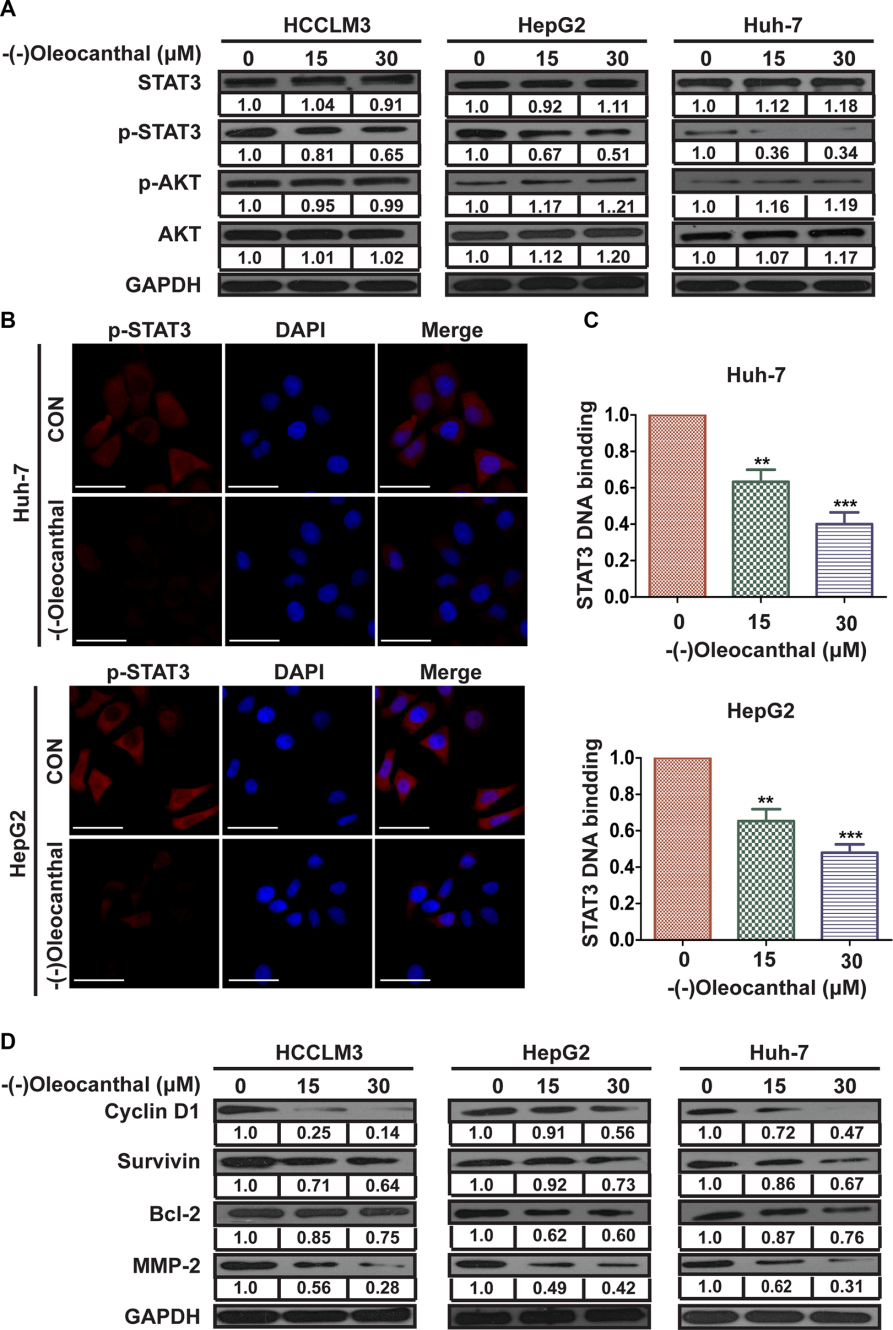
(−)-Oleocanthal suppresses the transcriptional activity of STAT3 and downregulates the expression of its target in HCC cells (**A**) Western blots analysis for the critical regulator of PI3K-AKT and STAT3 signal pathway in HCCLM3, HepG2 and Huh-7 cells. (**B**) (−)-Oleocanthal lead to the inhibition of translocation of p-STAT3 to the nucleus. HepG2 and Huh-7 cells were incubated with or without 15 μM for 12 h. Immunofluorescence was then used to analyze the intracelullar distribution of p-STAT3. Scale bar = 50 μm. (**C**) (−)-Oleocanthal inhibited STAT3 DNA-binding ability in HepG2 and Huh-7 cells. Cells were treated with (−)-oleocanthal for indicated dose; nuclear extracts were prepared for ELISA-based DNA-binding assay. (**D**) Western blots analysis for STAT3 and STAT3-regulated gene products in HCCLM3, HepG2 and Huh-7, with GAPDH as protein internal control. Data was presented as the means ± SD of three independent experiments. ** compared with control, *P* < 0.01. *** compared with control, *P* < 0.001.

To explore the mechanism by which (−)-oleocanthal inhibited the progression of HCC, we measured levels of critical regulators of the PI3K-AKT and STAT3 signal pathway in HCC. (−)-Oleocanthal decreased p-STAT3 levels without affecting p-AKT, total-AKT, or total-STAT3 levels in HCC cells (Figure 6A). (−)-Oleocanthal also reduced p-STAT5, but not STAT5, levels in HepG2 cells (Supplementary Figure S4). As nuclear translocation is critical for the function of transcription factors, we next determined the effect of (−)-oleocanthal on the nuclear translocation of STAT3 using immunofluorescence analysis. Nuclear p-STAT3 levels decreased in (−)-oleocanthal-treated HCC cells compared to control cells (Figure 6B). We then used an ELISA-based TransAM STAT3 assay kit to further explore whether (−)-oleocanthal suppressed the binding of STAT3 to DNA. (−)-Oleocanthal eliminated STAT3-DNA binding activity in Huh-7 and HepG2 cells (Figure 6C). We then measured levels of STAT3-regulated gene products using western blot. Levels of the cell cycle protein Cyclin D1, the anti-apoptotic proteins Bcl-2 and Survivin, and the invasion-related protein MMP-2 decreased after (−)-oleocanthal treatment in HCC cells (Figure 6D). Constitutive activation of STAT3 activates the Twist1 promoter [32]. To ascertain whether the decrease in Twist expression was due to an inhibition of Twist promoter activity, we generated a Twist1-promoter reporter construct (Twist-Luc) and performed a luciferase assay. (−)-Oleocanthal reduced Twist promoter activity in HepG2 cells in a dose-dependent manner (Supplementary Figure S5A). A Chromatin immunoprecipitation (CHIP) assay further confirmed that (−)-oleocanthal reduced the binding of STAT3 to the Twist gene promoter (Supplementary Figure S5B).

### (−)-Oleocanthal inhibits IL-6-induced activation of STAT3 and its anti-cancer effects are dependent on STAT3 expression

**Figure 7:**
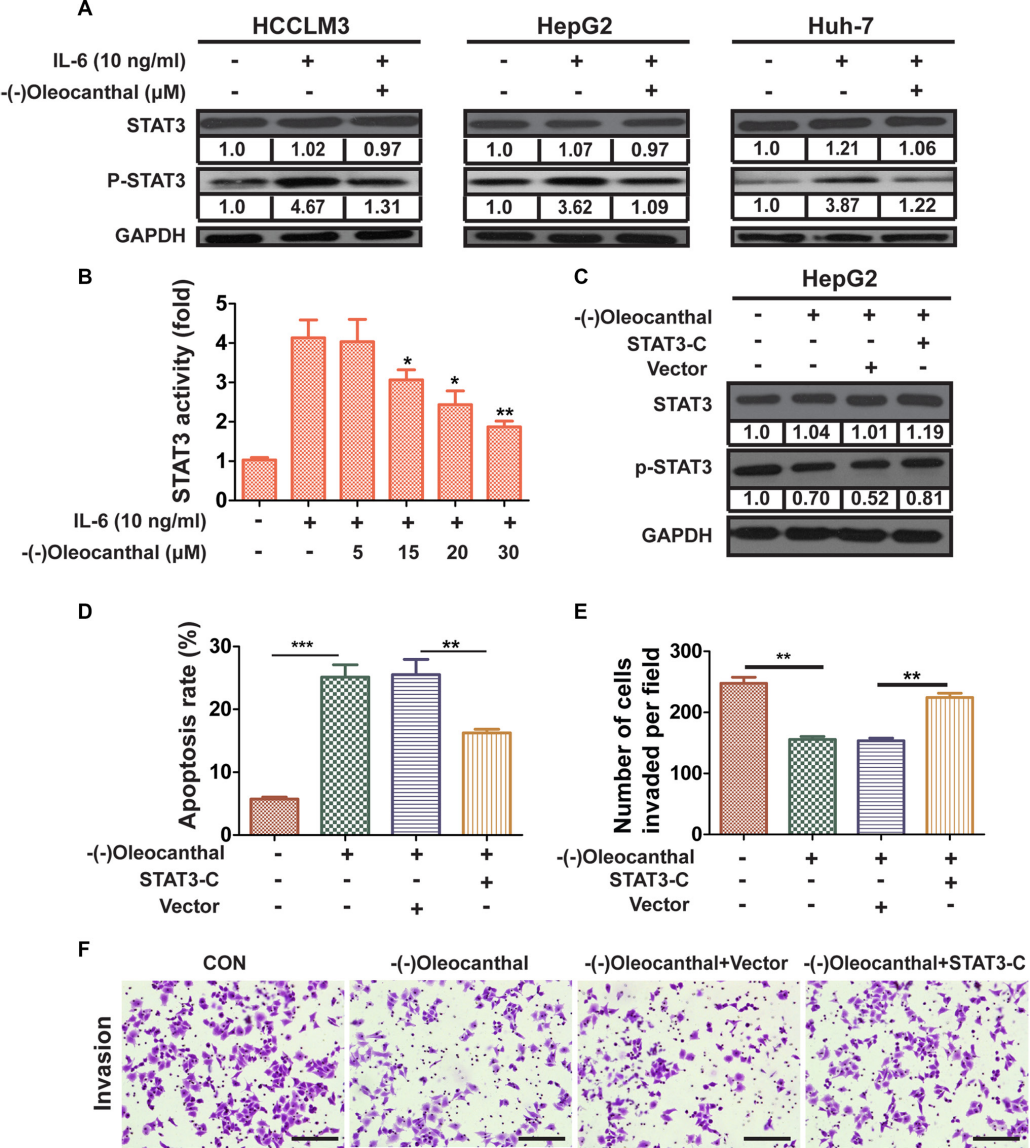
(−)-Oleocanthal inhibits IL6-inducible activation of STAT3 and its anti-cancer effects are dependent on STAT3 expression (**A**) HCCLM3, HepG2 and Huh-7 cells were treated with DMSO or (−)-oleocanthal (30 μM) for 24 h and then stimulated with IL-6 (10 ng/ml) for 4 h. The expression of p-STAT3 was detected by western blot. (**B**) HepG2 cells were transfected with STAT3-luciferase (STAT3-Luc) plasmid for 24 h, and treated with DMSO or (−)-oleocanthal (5, 15, 20, 30 μM) for 12 h and then stimulated with IL-6 (10 ng/ml) for 2 h. The transcriptional activity of STAT3 was measured by luciferase gene reporter assay. The measured luciferase activity was normalized to the activity of renilla luciferase. (**C**) HepG2 cells were transiently transfected with the vector control or the STAT3-C expression plasmid for 48 h and subsequently treated with (−)-oleocanthal (30 μM) for 24 h. Then, cells were harvested for western blots assay. (**D**) HepG2 cells was transfected with STAT3-C or vector for 48 hours. Cells were treated with (−)-oleocanthal (50 μM) for another 48 hours and cell apoptosis was tested by flow cytometry assay. (**E and F**) HepG2 cells was transfected with STAT3-C or vector for 48 hours. Cells were treated with (−)-oleocanthal (10 μM) for 24 hours and invasion assay was performed as abovementioned method. Representative images were shown and invaded cells were counted. Scale bar = 100 μm. Data was presented as the means ± SD of three independent experiments. * compared with control, *P* < 0.05. ** compared with control, *P* < 0.01. *** compared with control, *P* < 0.001.

We next investigated whether (−)-oleocanthal inhibited IL-6-induced activation of STAT3 in HCC cells. Pre-treatment with (−)-oleocanthal suppressed IL-6-induced STAT3 activation in HCC cells (Figure 7A). IL-6 increased STAT3-mediated luciferase gene expression in control HepG2 cells; however, pre-treatment with (−)-oleocanthal suppressed IL-6-induced STAT3 activation in a dose-dependent manner (Figure 7B). To further investigate whether STAT3 inhibition was necessary for (−)-oleocanthal-induced suppression of HCC progression, HepG2 cells were treated with (−)-oleocanthal following transient transfection with constitutively active STAT3 (STAT3-C) (Figure 7C). Overexpression of STAT3-C partly blocked the effects of (−)-oleocanthal on apoptosis and invasion in HepG2 cells (Figure 7D, 7E and 7F). Furthermore, the STAT3-specific inhibitor NSC 74859 enhanced the anti-migratory and anti-invasive effects of (−)-oleocanthalin HepG2 cells (Supplementary Figure S6A–S6C).

### (−)-Oleocanthal inhibits STAT3 activation by altering the activity of positive and negative STAT3 regulators

**Figure 8:**
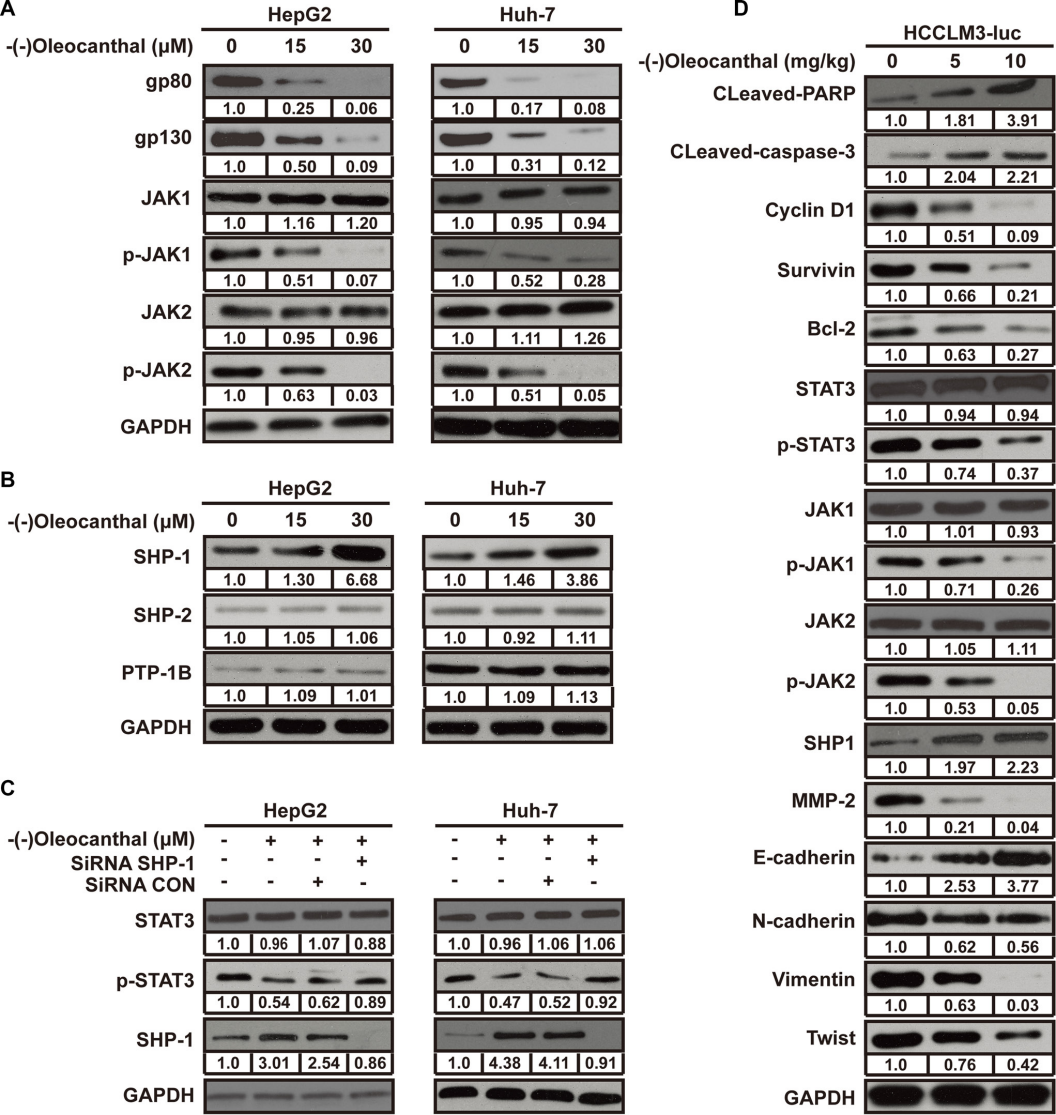
(−)-Oleocanthal inhibits the activation of STAT3 through regulating the expression of positive and negative regulators (**A**) Western blots analysis for the positive regulator of STAT3 in HepG2 and Huh-7 cells. (**B**) Western blots analysis for the negative regulator of STAT3 in HepG2 and Huh-7 cells. (**C**) HepG2 and Huh-7 cells were transfected with either SHP-1 siRNA or scrambled siRNA. After 48 h, cells were treated with 30 μM of (−)-oleocanthal for 48 h and whole cell extracts were subjected to Western blot analysis for phosphorylated STAT3. (**D**) Western blot analysis was performed to detect the expression of indicated protein in tumor tissue.

To explore the mechanism by which (−)-oleocanthal decreased p-STAT3 levels, we first measured levels of two positive STAT3 regulators (JAK1 and JAK2). (−)-Oleocanthal reduced levels of p-JAK1, p-JAK2, gp80, and gp130, but did not affect total-JAK1 or total-JAK2 levels (Figure 8A). We then measured levels of several negative STAT3 regulators (SHP1, SHP-2, and PTP-1B). (−)-Oleocanthal upregulated SHP-1 protein levels, but did not affect SHP-2 or PTP-1B levels (Figure 8B). After SHP-1 expression was silenced using small-interfering RNA (siRNA), the (−)-oleocanthal-induced decrease in p-STAT3 levels was partly reversed (Figure 8C). To determine whether (−)-oleocanthal inhibited STAT3 activity in nude mouse tumor tissues, we measured relative levels of the proteins mentioned above using western blot. The results were similar to those from the *in vitro* experiments (Figure 8D).

## DISCUSSION

(−)-Oleocanthal is a phenolic compound first discovered in VOO in the early 90′s. Previous studies have reported that (−)-oleocanthal has anti-oxidation, anti-bacterial, and anti-inflammation effects and acts as a COX inhibitor [21–25]. (−)-Oleocanthal exerts anti-tumor effects by regulating key tumor-related signal pathways [29–31]. Here, we demonstrated for the first time that (−)-oleocanthal inhibited HCC growth and metastasis both *in vitro* and *in vivo*, at least in part by inhibiting the activity of STAT3 and its gene products.

Many previous studies have shown that (−)-oleocanthal inhibits tumor growth. Khanal etal. demonstrated that (−)-oleocanthal treatment suppresses the proliferation and malignant transformation of mouse epidermal JB6 Cl41 cells by inhibiting the ERK pathway [33]. In human breast cancer MDA-MB-231 cells, (−)-oleocanthal induces G1/M arrest by modulating the expression of CDK6, cyclin D1, p21, and p27 and induces cell apoptosis by activating the caspase pathway [29]. Here, we found that (−)-oleocanthal inhibited proliferation by inducing cell cycle arrest and apoptosis in HCC cells both *in vitro* and *in vivo*. Cleavage of PARP and caspase-3 was also upregulated after (−)-oleocanthal treatment. Because previous research has shown that (−)-oleocanthal inhibits metastasis of some cancers [30], we next explored its effect on metastasis of HCC. (−)-Oleocanthal indeed suppressed HCC migration, invasion, and metastasis *in vitro* and *in vivo*. In addition, the same dose of (−)-oleocanthal had no effect on the viability of normal liver LO2 cells, suggesting that it inhibited HCC progression specifically with minimal side effects.

To investigate the specific mechanism by which (−)-oleocanthal inhibited HCC growth and metastasis, we examined two signal pathways (STAT3 and PI3K/AKT) related to HCC growth and metastasis. (−)-Oleocanthal decreased p-STAT3 levels, but did not affect STAT3, p-AKT, or AKT levels. (−)-Oleocanthal inhibited STAT3 activation by downregulating STAT3 nuclear translocation and DNA binding activity in HCC cells. (−)-Oleocanthal also decreased levels of the cell cycle protein Cyclin D1, the anti-apoptotic proteins Bcl-2 and Survivin, and the invasion-related protein MMP-2, which are regulated by STAT3. More importantly, STAT3-C overexpression partially reversed the (−)-oleocanthal-induced increase in apoptosis and inhibition of invasion in HCC cells, indicating that its anti-cancer effects were partly dependent on STAT3 activation. We then explored the mechanism by which (−)-oleocanthal inhibited STAT3 activation. IL-6 can activate several tyrosine kinases (JAK1, JAK2 or c-Src) that act through specific receptors to activate STAT3 via phosphorylation [6–8]. In contrast, STAT3 activity is negatively regulated by suppressor of cytokine signaling proteins (SOCS) 1/3, cytokine-inducible SH2-containing (CIS) protein, and protein tyrosine phosphatases (PTPases) [34–37]. We found that (−)-oleocanthal suppressed IL-6-induced STAT3 activation. Furthermore, (−)-oleocanthal decreased levels of the positive STAT3 regulators p-JAK1 and p-JAK2 and increased levels of the negative STAT3 regulator SHP-1 to inhibit STAT3 activation.

The role of EMT in cancer progression remains a topic of debate. EMT may initially facilitate invasion and dissemination of cancer cells, which then undergo an opposing process, termed mesenchymal–epithelial transition (MET), that increases clonal outgrowth at metastatic sites [38]. However, recent evidence suggests that EMT is unnecessary for breast cancer and pancreatic cancer metastasis [39, 40]. Because cancer is a heterogeneous disease, it remains to be determined whether these results are generalizable to other cancer types. It should also be noted that, even if EMT is not necessary for metastasis, it may still contribute to the process. Studies by Fischer et al. and Zheng et al. show that EMT does confer chemoresistance [39, 40], suggesting that inhibition of EMT may still be an important mechanism for suppressing cancer progression. Here, (−)-oleocanthal inhibited EMT by altering both protein levels and mRNA expression. We further examined the expression of several transcriptional factors that induce EMT by suppressing E-cadherin expression. Among these, (−)-oleocanthal reduced Twist protein levels and mRNA expression. It has been reported that STAT3 influences EMT phenotypes by modulating the expression of “master” EMT transcriptional factors, including Twist. Moreover, Twist is a direct target of STAT3. Therefore, a CHIP assay was performed and showed that (−)-oleocanthal reduced the binding of STAT3 to the Twist gene promoter.

**Figure 9:**
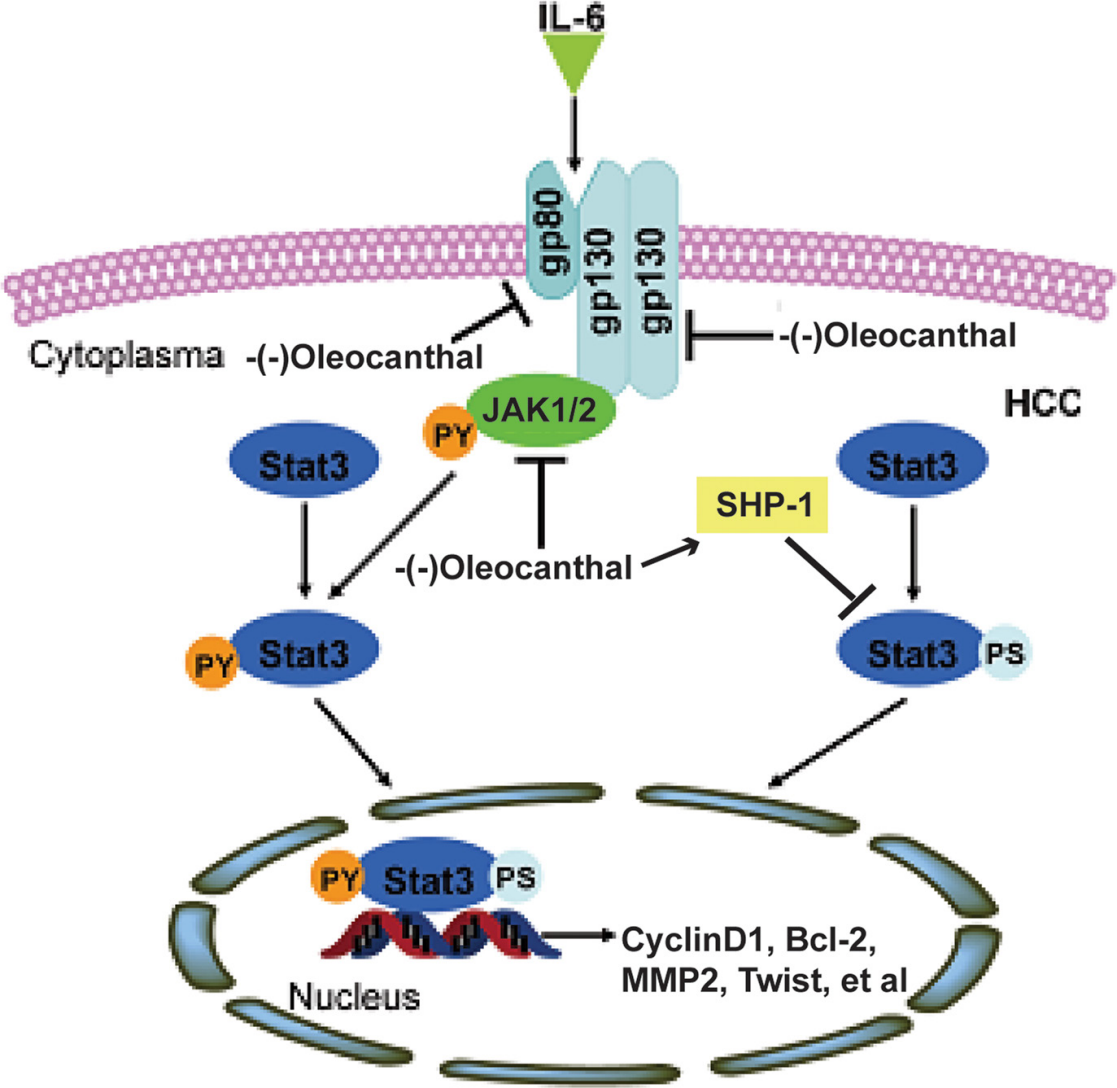
A schematic model for the role of (−)-oleocanthal in HCC

In summary, as outlined in Figure 9, we have shown that (−)-oleocanthal inhibits HCC tumor growth and metastasis by inactivating STAT3 both *in vitro* and *in vivo*. Although clinical trials are needed to confirm these findings, (−)-oleocanthal may be useful in HCC prevention and treatment.

## MATERIALS AND METHODS

### Cell lines and reagents

The immortalized LO2 liver cell line and HCCLM3 and Huh-7 liver cancer cell lines were purchased from the Cell Bank of Type Culture Collection of the Chinese Academy of Sciences at the Shanghai Institute of Cell Biology. The HepG2 cell line was obtained from the American Type Culture Collection (Manassas, VA). All cells were cultured in Dulbecco's modified Eagle medium (DMEM) supplemented with 10% fetal bovine serum and antibiotics (100 U/mL penicillin and 100 μg/mL streptomycin). IL-6 was purchased from Sigma. Lipofectamine 2000 was obtained from Invitrogen. Rabbit monoclonal antibodies against phospho-specific JAK2 (Tyr 1007/1008), JAK2, phospho-specific JAK1 (Tyr 1022/1023), JAK1, phospho-STAT3 (Tyr 705), STAT3, AKT, phospho-AKT, ERK, cleaved-PARP, cleaved-caspase-3, Bcl-2, survivin, SHP-1, SHP-2, PTP-1B, E-cadherin, and Twist were obtained from Cell Signaling Technology (Danvers, MA). N-cadherin, Vimentin, and Ki-67 antibodies were purchased from Abcam (Cambridge, MA). MMP2 antibody, anti-mouse IgG-HRP, and anti-rabbit IgG-HRP were purchased from Santa Cruz Biotechnology (Santa Cruz, CA). siRNA for SHP1 (sc-44101) and scrambled control (sc-37007) was obtained from Santa Cruz Biotechnology (Santa Cruz, CA). STAT3-C plasmid was constructed by GeneChem Corporation (Shanghai, China); substitution of two cysteine residues within the C-terminal loop of the STAT3 SH2 domain generates a constitutively active STAT3 [41].

### Extraction and isolation of (−)-oleocanthal

Approximately 2 L of n-hexane and 1 kg of EVOO (Sigma-Aldrich, MO) were mixed, CH_3_CN-MeOH (1 L, 20:80) was added, and the mixture was shaken twice. The dried organic layer (24 g) was subjected to repeated medium pressure liquid chromatography (MPLC) in a 50 × 3 cm column with lipophilic Sephadex LH20 (bead size 25−100 μm, Sigma-Aldrich, MO) using n-hexane-CH_2_Cl_2_ (1:9) and isocratic elution followed by MPLC (10 g, 25×1 cm column) with C-18 reversed-phase silica gel and Bakerbond octadecyl (40 μm; Mallinckrodt Baker, Inc.), yielding 13.3 mg of > 97% pure (−)-oleocanthal (measured using HLPC) and several other impure fractions. Identification and determination of purity were also based on comparison of ^1^H and ^13^C NMR data to the literature [42]. Generally, 1:100 ratios of mixtures to be chromatographed to stationary phase were used in all liquid chromatographic purifications.

### Establishment of the stable HCCLM3-luciferase cell line

Lentiviral vectors encoding the human fireflyLuciferase gene were constructed by GeneChem Corporation (Shanghai, China). The empty vector was used as a negative control. The lentiviral vectors were transfected into HCCLM3 cells with a multiplicity of infection (MOI) of 60 in the presence of polybrene (5 μg/mL). 48 hours after transfection, transfected cells were selected by treatment with 2.5 μg/mL puromycin (Sigma) for 2 weeks. Cells obtained 2 weeks after drug selection without subcloning were used in experiments.

### Cell viability assay

Cell viability was determined using a Cell Counting Kit-8 (CCK-8) kit (Dojindo Laboratories, Kumamoto, Japan). Briefly, HCC cells were incubated in a 96-well plate at adensity of 2500 per well and treated with increasing doses of (−)-oleocanthal (0-80 μM, dissolved in DMEM) for 24–72 h. After treatment, cell culture supernatant was discarded, 100 μL DMEM containing 10 μL CCK-8 solution was added to each well, and the 96-well plate was incubated at 37°C for 2 h. The absorbance of each well at 450 nm was measured using a microplate reader.

### Cell cycle analysis

Cell cycle analysis was performed as previously described [43]. HCC cells were treated with (−)-oleocanthal for 48 hours followed by PBS washes. Cells were then fixed with cold 70% ethanol at 4°C for at least 12 hours. PI working solution was added before flow cytometry analysis (FACSCalibur, BD Biosciences).

### Apoptosis assay

Apoptosis was detected using the Apoptosis Detection Kit (BD Biosciences) as previously described [43]. HCC cells were treated with (−)-oleocanthal for 48 hours, then collected, washed, and stained with annexin V–FITC and PI for 15 minutes before evaluation by flow cytometry (FACSCalibur, BD biosciences).

### TUNEL assay

Apoptosis was detectedusing the TUNEL enzyme kit (Roche, Shanghai, China) according to manufacturer's instructions.

### Migration and invasion assay

Cell motility and invasive ability were investigated using transwell migration (BD Biosciences, San Jose, CA, USA) and Matrigel invasion (BD Biosciences) assays, respectively. 2.5–3 × 10^4^ and 3–4 × 10^4^ cells were seeded for the migration and invasion assays, respectively. The assays were performed for 24 h after 24 h of pre-treatment with different doses of (−)-oleocanthal. Cells that migrated to the underside of the membrane were fixed and stained with 0.5% crystal violet and then counted under an optical microscope.

### Wound-healing assay

The wound-healing assay was performed as described previously [43]. Briefly, 5–6 × 10^5^ HCC cells per well were seeded in a 6-well plate and allowed to grow to confluence. Cells were then washed three times in medium, scratched with a 10 μL pipette tip, and incubated in growth medium with 15 μM (−)-oleocanthal. Images were taken after 0 h, 24 h, and 48 h of incubation at 10× on a Nikon Eclipse TS100 microscope.

### Western blot

Western blots were performed as described previously [43]. Briefly, whole-cell extracts were sonicated in lysis buffer and homogenized. Samples containing 30-50 μg of total protein were resolved on 8–12% polyacrylamide SDS gels and electrophoretically transferred topolyvinylidene difluoride (PVDF) membranes. The membranes were blocked with 5% skim milk, incubated with primary antibody, and subsequently incubated with an alkaline phosphatase-conjugated secondary antibody. Detection of specific proteins was performed using ECL reagents (Pierce, Rockford, IL).

### Quantitative real-time PCR

RNA isolation and quantitative real-time PCR were performed as described previously [44]. HCC cells were harvested by brief centrifugation after treatment with (−)-oleocanthal and pellets were stored for RNA isolation. Primer sequences were as follows:

Snai1 primer: sense, 5′-TTACCTTCCAGCAGCC CTAC-3′, anti-sense, 5′-AGCCTTTCCCACTGTCCTC-3′; Twist primer: sense, 5′-CGACGACAGCCTGAGCAACA-3′, anti-sense, 5′-CCACAGCCCGCAGACTTCTT-3′; Slug primer: sense, 5′-CCTCCATCTGACACCTCC-3′, anti-sense, 5′-CCCAGGCTCACATATTCC-3′; Zeb1 primer: sense, 5′-AAGTGGCGGTAGATGGTA-3′, anti-sense, 5′-TTGTAGCGACTGGATTTT-3′;SIP1 primer: sense, 5′-TTCTGCGACATAAATACG-3′, anti-sense, 5′-GAGTGAAGCCTTGAGTGC-3′;E-cadherin primer: sense, 5′-TGCCCAGAAAATGAAAAAGG-3′, anti-sense, 5′-GTGTATGTGGCAATGCGTTC-3′; N-cadherin primer: sense, 5′- AGGATCAACCCCATACACCA-3′, anti-sense, 5′- TGGTTTGACCACGGTGACTA-3′;Vimentin primer: sense, 5′- GAGAACTTTGCCGTTGAAGC-3′, anti-sense, 5′- TCCAGCAGCTTCCTGTAGGT-3′; GAPDH primer: sense, 5′-GCACCGTCAAGGCTGAGAAC-3′, anti-sense, 5′-TGGTGAAGACGCCAGTGGA-3′.

### Transfection with SHP-1 siRNA

siRNA knockdown of SHP-1 was performed as previously described [43]. Briefly, HCC cells were plated onto a six-well plate and allowed to adhere overnight. Then, HCC cells were transiently transfected with the SHP-1-siRNA or the non-targeting scrambled siRNA for 48 hand used for subsequent experiments.

### DNA binding assay

The DNA binding assay was performed using the TransAM STAT3 transcription factor assay kit according to the manufacturer's instructions.

### Luciferase reporter assay

The luciferase reporter assay was performed as described previously [45]. Luciferase activity was determined using the Promega luciferase assay kit.

### Immunohistochemistry analysis

Immunohistochemistry was performed as previously described [43]. Primary tumors were excised, fixed, and embedded in paraffin. Then, sections were stained with Ki-67 and CD31 antibodies. Images were obtained with a Leica microscope (Leica, DM4000b). Results were analyzed using Image-Pro Plus 6.0 software.

### Immunofluorescence assay

Briefly, cells seeded on cover slips were fixed with 4% (w/v) paraformaldehyde (Sigma) for 10 min and permeabilized with 0.1% (v/v) Triton X-100 for 5 min at room temperature. The cells were then incubated with primary antibodies at 4°C overnight. Cells were incubated with fluorescent secondary antibody (Invitrogen) for 1 hour at room temperature on the second day. Cells were then washed with PBS and coverslips were mounted using an anti-fade mounting solution containing 4′,6-diamidino-2-phenylindole (DAPI; Vector lab, Burlingame, CA) before image acquisition and analysis.

### Chromatin immunoprecipitation (CHIP) assay

The CHIP assay was performed as described previously [43]. Briefly, HepG2 cells were treated with 15 μM (−)-oleocanthal for 12 hours. The cells were cross-linked by the addition of formaldehyde at a 1% final concentration, chromatin was sonicated, and immunoprecipitation was performed using 1 μg of STAT3, c-fos and IgG antibody. The CHIP assay was performed using a commercially available CHIP assay kit (Simple CHIP, Cell Signaling Technology) according to the manufacturer's instructions. Twist mRNA levels were evaluated using RT-PCR. Human Twist promoter sequences were detected in the immunoprecipitates using PCR with the following primers: forward, 5′-AGTCTCCTCCGACCGCTTCCTG-3′, reverse, 5′-CTC CGTGCAGGCGGAAAGTTTGG -3′.

### Orthotopic animal model of HCC

Male BALB/c athymic nude mice (4–6 weeks old) were obtained from the experimental animal center of the Shanghai Institute for Biological Sciences (SIBS) and housed under standard conditions according to the institutional guidelines for animalcare. All of the animal experiments were approved by the Institutional Animal Care and Use Committee of Harbin Medical University. To establish the orthotopic hepatocellular carcinoma mouse model, 4 × 10^6^ HCCLM3-luc cells in 150 μL of phosphate-buffered saline were subcutaneously injected into the flanks of nude mice. After 1 week, the subcutaneous tumors were excised and diced into 1 mm^3^ cubes, which were then implanted into the left lobes of the livers of the mice. Once increasing bioluminescence tumor signals were detected in the mice, they were randomly separated into three groups (*n* = 6). Mice were treated with (−)-oleocanthal (5 mg/kg/d or 10 mg/kg/d, i.p.) for five weeks. The control group received injections of DMSO. Tumor growth was monitored using the bioluminescence IVIS Imaging System. For imaging, mice were given i.p. injections of 100 mg/kg D-luciferin (Xenogen, Hopkinton, MA) 5 min before imaging. At the end of the treatment, animals were euthanized and tumors were harvested for subsequent analysis.

### Establishment of orthotopic HCC patient-derived xenografts

HCC tissues were collected from HCC patients who had undergone liver resection as part of their treatment. The use of all samples was approved by the Committees for Ethical Review of Research at the First Affiliated Hospital of Harbin Medical University. HCC specimens were mechanically and enzymatically dissociated in HBSS containing 0.1% collagenase, 0.01% hyaluronidase, and 0.002% deoxyribonuclease at 37°C to obtain single cell suspensions. Cells were then passed through a 70-μm filter, centrifuged at 100 g for 10 minutes, resuspended in Freezing Medium (FBS containing 10% DMSO) for storage at −80°C overnight, and transferred to liquid nitrogen for long-term storage. Thawed cells were resuspended in BEGM medium mixed with 50% Matrix Matrigel (Becton Dickinson; Franklin Lakes, NJ) and injected subcutaneously into male BALB/c athymic nude mice (5 weeks old, *n* = 6). After 1 week, the subcutaneous tumors were excised and diced into 1 mm^3^ cubes, which were then implanted into the left lobes of the livers of the mice. Mice were treated with (−)-oleocanthal (5 mg/kg/d or 10 mg/kg/d, i.p.) for five weeks. The control group received DMSO injections. At the end of the treatment, the mice were euthanized and tumor volumes were calculated using the following equation: tumor volume = length × (width)^2^ × π/6.

### *In vivo* experimental metastasis assay

BALB/c mice were acquired and raised after obtaining appropriate institutional review board permission as described above. To establish the experimental metastasis model, 8 mice in each group were given tail vein injections of HCCLM3-luc cells (3 × 10^6^). Mice were treated with (−)-oleocanthal (5 mg/kg/d or 10 mg/kg/d, i.p.) for eight weeks. The control group received DMSO injections. Tumor metastases were imaged and quantified using bioluminescencen every two weeks after the fourth week. At the end of the treatment, mice were sacrificed and tumor nodules on the lungs were counted. Lungs were excised to perform further experiments.

### Statistical analysis

Results are presented as mean values ± standard deviation (SD). Comparisons between multiple groups were performed using one-way analysis of variance(ANOVA) followed by Dunnett'stest. A value of *P* < 0.05 was considered statistically significant.

## SUPPLEMENTARY MATERIAL FIGURES


