# Supplementary material for: (−)-Oleocanthal inhibits growth and metastasis by blocking activation of STAT3 in human hepatocellular carcinoma
Source: Oncotarget. 2016 Jun 2;7(28):43475–91. doi: 10.18632/oncotarget.9782 (PMC5190038; doi:10.18632/oncotarget.9782)
Supplement: Supplementary file 1 [file oncotarget-07-43475-s001.pdf]

(-)-Oleocanthal inhibits growth and metastasis by blocking activation of STAT3 in human hepatocellular carcinoma

Supplementary Materials

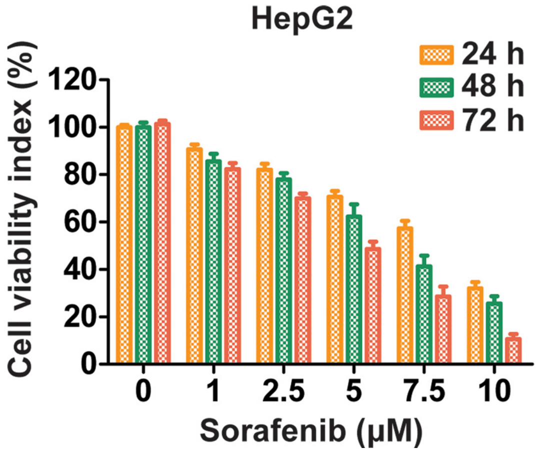

Supplementary Figure S1: Sorafenib inhibits proliferation of HepG2 cells *in vitro*. HepG2 cells were incubated with increasing doses of Sorafenib (0–10 μM) for 24–72 h. CCK-8 assays were then performed to determine the cell viability index. Results are shown as the means ± SD of experiments performed in triplicate.

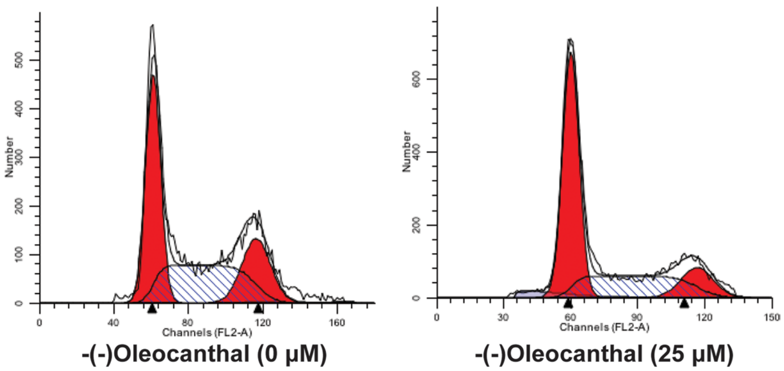

Supplementary Figure S2: (-)-Oleocanthal inhibits cell cycle progression in HCC cells *in vitro*. A representative image of cell cycle arrest in HepG2 cells after 48 h of treatment with 25 μM of (-)-oleocanthal is shown.

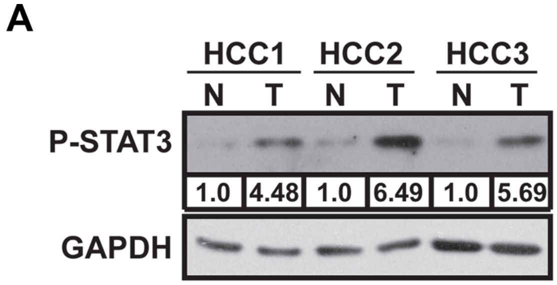

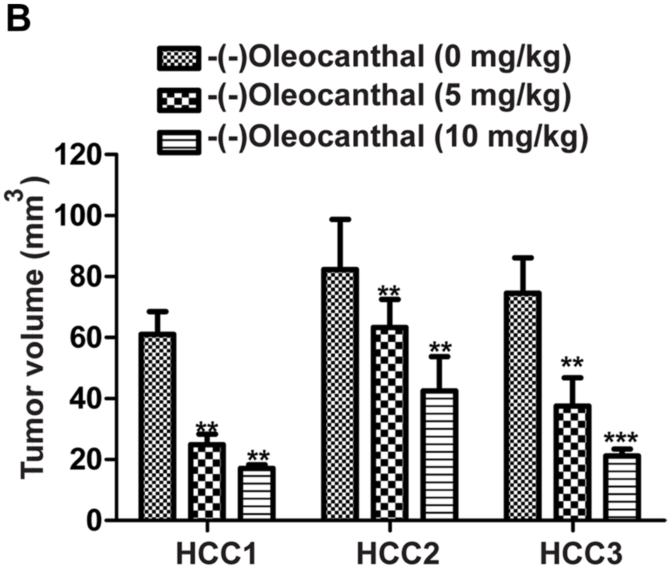

C

	Age(y)	Gender	Race	Virus
HCC-1	47	Male	Asian	HBV
HCC-2	51	Female	Asian	HBV
HCC-3	63	Male	Asian	None

Supplementary Figure S3: (-)-Oleocanthal inhibits growth of orthotopic HCC patient-derived xenografts. (**A**) GAPDH (loading control) and p-STAT3 protein levels in tumor (T) and matched non-tumor liver (N) tissues from three HCC patients were determined using western blot. (**B**) Tumor volumes of HCC-1, HCC-2, and HCC-3 xenografts were measured with vernier calipers after 5 weeks of treatment with (-)-oleocanthal. (**C**) Characteristics of the three HCC patients from whom tissues were obtained. Data are shown as the means ± SD of three independent experiments. $^{**}P < 0.01$ and $^{***}P < 0.001$ compared to controls.

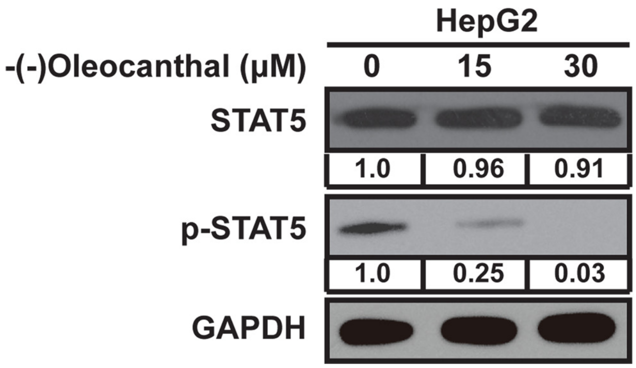

Supplementary Figure S4: Western blot assay for STAT5 and p-STAT5 in HepG2 cells after (-)-oleocanthal treatment. HepG2 cells were treated with 15 or 30 μM (-)-oleocanthal for 48 h.

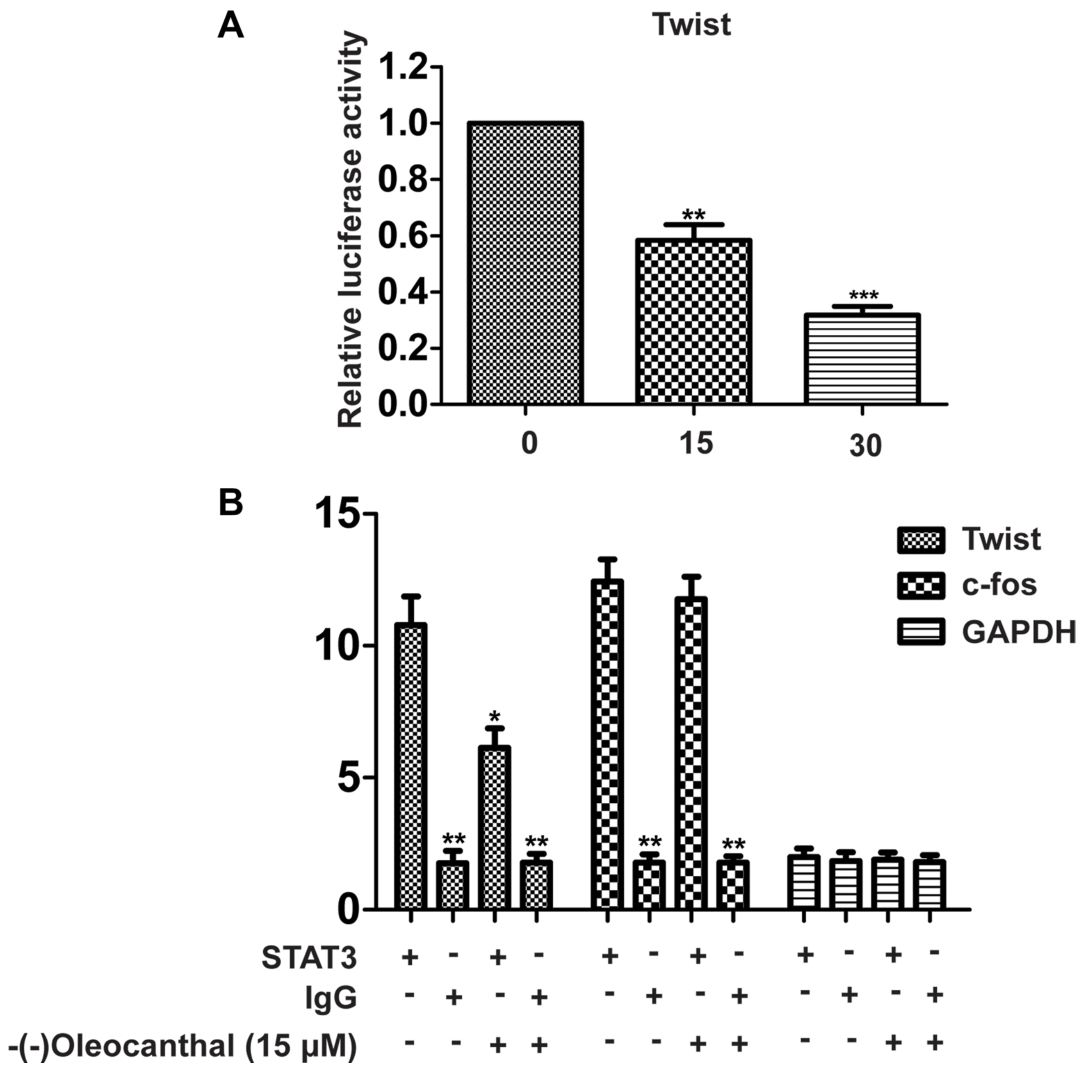

Supplementary Figure S5: **(-)-Oleocanthal inhibits the binding of STAT3 to the Twist gene promoter in HepG2 cells.** (**A**) HepG2 cells were transfected with a Twist luciferase plasmid for 12 h and then exposed to various concentrations of (-)-oleocanthal for another 12 h. The transcriptional activity of Twist was measured using a luciferase gene reporter assay. Luciferase activity was normalized to renilla luciferase activity. (**B**) ChIP was performed to detect the interaction between STAT3 and the Twist promoter using anti-STAT3 and anti-IgG antibodies. GAPDH and the known STAT3 target c-fosare shown as negative and positive controls, respectively. Data are shown as the means ± SD of three independent experiments. *$P < 0.05$,**$P < 0.01$, and ***$P < 0.001$ compared to controls.

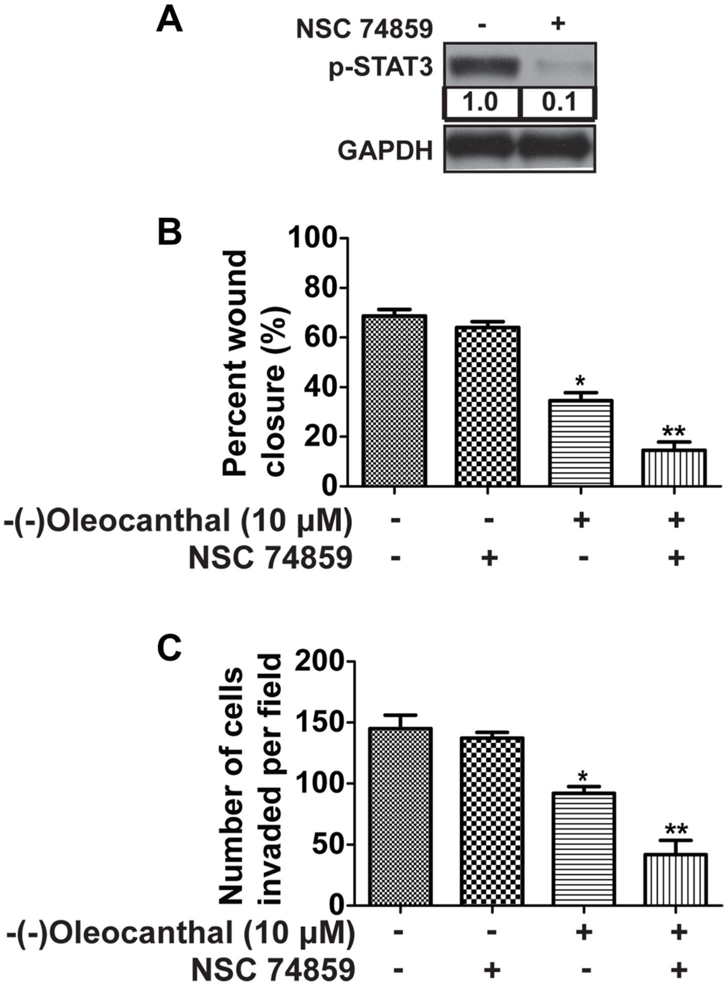

Supplementary Figure S6: NSC 74859 enhanced the anti-migratory and anti-invasive effects of (-)-oleocanthal in HepG2 cells. (**A**) Western blot assay for p-STAT3 in Huh-7 cells after NSC 74859 (50 μM) treatment. (**B**) Huh-7 cells were treated with (-)-oleocanthal (10 μM) or NSC 74859 (50 μM) for 48 hours; wound closure was then quantified. (**C**) Huh-7 cells were treated with (-)-oleocanthal (10 μM) or NSC 74859 (50 μM) for 24 hours; invaded cells were then counted in an invasion assay. Data are shown as the means ± SD of three independent experiments. **P* < 0.05 and ***P* < 0.01 compared to controls.
